# The Repeat Region of the Circumsporozoite Protein is Critical for Sporozoite Formation and Maturation in *Plasmodium*

**DOI:** 10.1371/journal.pone.0113923

**Published:** 2014-12-01

**Authors:** David J. P. Ferguson, Amanda E. Balaban, Eva-Maria Patzewitz, Richard J. Wall, Christine S. Hopp, Benoit Poulin, Asif Mohmmed, Pawan Malhotra, Alida Coppi, Photini Sinnis, Rita Tewari

**Affiliations:** 1 Nuffield Department of Clinical Laboratory Science, University of Oxford, John Radcliffe Hospital, Oxford, OX3 9DU, United Kingdom; 2 Department of Molecular Microbiology & Immunology, Johns Hopkins Bloomberg School of Public Health, Baltimore, Maryland, United States of America; 3 Centre for Genetics and Genomics, School of Life Sciences, Queens Medical Centre, University of Nottingham, Nottingham, NG2 7UH, United Kingdom; 4 International Centre for Genetic Engineering and Biotechnology, New Delhi-110067, India; INSERM, France

## Abstract

The circumsporozoite protein (CSP) is the major surface protein of the sporozoite stage of malaria parasites and has multiple functions as the parasite develops and then migrates from the mosquito midgut to the mammalian liver. The overall structure of CSP is conserved among *Plasmodium* species, consisting of a species-specific central tandem repeat region flanked by two conserved domains: the NH_2_-terminus and the thrombospondin repeat (TSR) at the COOH-terminus. Although the central repeat region is an immunodominant B-cell epitope and the basis of the only candidate malaria vaccine in Phase III clinical trials, little is known about its functional role(s). We used the rodent malaria model *Plasmodium berghei* to investigate the role of the CSP tandem repeat region during sporozoite development. Here we describe two mutant parasite lines, one lacking the tandem repeat region (ΔRep) and the other lacking the NH_2_-terminus as well as the repeat region (ΔNΔRep). We show that in both mutant lines oocyst formation is unaffected but sporozoite development is defective.

## Introduction

Malaria is caused by apicomplexan protozoan parasites of the genus *Plasmodium* and is responsible for approximately 1 million deaths per year [1]. The *Plasmodium* life cycle is complex and alternates between vertebrate and mosquito hosts. In susceptible mammals, the disease is transmitted by the bite of an infected female *Anopheles* mosquito. As the mosquito probes for blood, sporozoites, the infective stage of the parasite, are injected into the skin of the mammalian host. From the injection site in the dermis, sporozoites move actively by gliding motility to locate a blood vessel, which they penetrate to enter the blood circulation. Once in the blood stream, sporozoites reach the liver, where they arrest and invade hepatocytes after crossing the sinusoidal barrier. Inside hepatocytes they develop into exo-erythrocytic forms, which release merozoites to initiate blood stage infections [2]. The asexual blood stages are responsible for the clinical manifestations of the disease. A small proportion of asexual parasites develop into sexual stage gametocytes that are ingested with the blood upon mosquito feeding. Sexual reproduction of the parasite occurs in the mosquito midgut, leading to the development of ookinetes, which penetrate the midgut wall and develop into oocysts on the basal surface of the midgut. Thousands of sporozoites develop in individual oocysts and when mature, egress into the hemocoel to invade the salivary glands and begin the cycle anew.

Previous studies have demonstrated that inoculation of irradiated sporozoites can induce sterile immunity to malaria infection in both humans and animals [3]–[5]. One of the targets of this protective immune response is the major surface protein of the sporozoite, the circumsporozoite protein (CSP). Antibodies, specific for the central repeat region of CSP, and T cells, recognizing epitopes in the carboxy-terminus of CSP, are the central components of this immunity (reviewed in [6]). RTS, S, a subunit malaria vaccine candidate composed of the CSP repeats and the TSR domain fused to the hepatitis B surface antigen has shown promise in Phase III clinical trials [7], [8], validating the CSP repeats as a vaccine target.

CSP is a multifunctional protein, forming a dense coat on the surface of the sporozoite. Its overall structure is highly conserved in all *Plasmodium* species, consisting of a central repeat region flanked by an NH_2_-terminal domain containing a conserved proteolytic cleavage site, and a COOH-terminal cell-adhesion domain, the thrombospondin repeat (TSR) [9]–[12]. Deletion of the *csp* gene gives rise to oocysts in which sporozoites do not develop, demonstrating a critical role for this protein in sporozoite development [13], [14]. Various studies have dissected the functional role of the NH_2_ and COOH-terminal regions during egress from oocysts, invasion of salivary glands, exit from the inoculation site and localization to and invasion of hepatocytes [9], [12], [15]–[18]. After their release from oocysts, the NH_2_-terminus of CSP mediates adhesion to salivary glands [19] and in the mammalian host, it masks the TSR, maintaining the sporozoite in a migratory state [15]. Once in the liver, a regulated proteolytic cleavage event leads to the removal of the NH_2_-terminal third of the protein exposing the TSR [9], [15], [20], an event that is critical for efficient invasion of hepatocytes by sporozoites.

Despite the large number of studies investigating the structure and function of CSP, only a limited number of studies have addressed the function of the central repeat region. Although the sequence of the repeats varies among *Plasmodium* species, the relatively small pool of amino acids present in the repeats, as well as their similar length, suggest structural and/or functional constraints. Studies in which the CSP repeats of the rodent parasites have been replaced by those from other species demonstrate no difference in sporozoite infectivity, supporting the conservation of function among different species [21]–[23]. Given the importance of the repeats regions as a target of protective antibodies, we used a genetic approach to analyze the function of the CSP repeats. The results presented here suggest that the repeat region of CSP plays a critical role in sporozoite development in the mosquito vector.

## Results

### Generation of mutant parasites lacking the central repeat region of CSP

**Figure 1:**
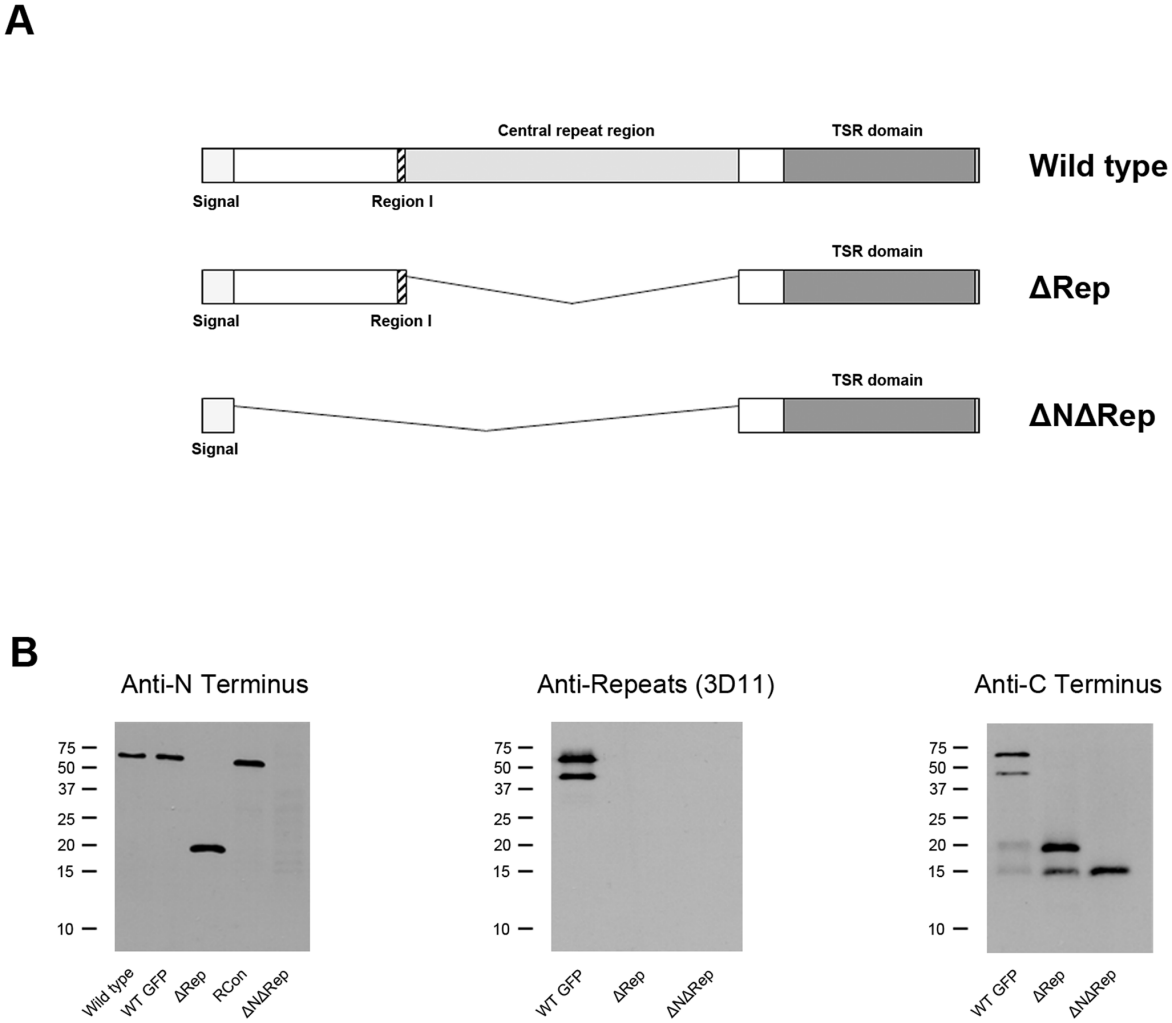
Generation of CSP repeatless mutants. **A.** Schematic representation of CSP structure in wild type and mutant parasites ΔRep and ΔNΔRep. Region I is shown as hatched, repeat region as light grey and the TSR domain as dark grey. **B.** Western blot analysis of wild type (WT), WT-GFP and RCon as control parasites and the two repeat mutants: ΔRep and ΔNΔRep. Lysates from midgut sporozoites or infected midguts were probed using antisera specific for each of the three CSP domains: polyclonal antisera specific for the CSP NH_2_-terminus, anti-repeat region (mAb 3D11) and polyclonal antisera specific for the CSP COOH-terminus. Molecular weight markers (kDa) shown on the left of each gel photograph.

To dissect the function of the central repeat region of CSP, we generated two mutant parasite lines in which we replaced the endogenous *csp* locus with a mutant version lacking either the repeat region, ΔRep, or lacking both the NH_2_-terminus and the repeat region, ΔNΔRep (Figure 1A). Transfection constructs included homologous upstream and downstream sequence to direct double homologous recombination and replacement of the endogenous *csp* gene (Figures S1 and S2). *P. berghei* ANKA parasites expressing GFP under the ef1alpha promoter (507cl1) were used as the parent clone for ΔRep mutant generation [24] and wild type *P. berghei* ANKA parasites were used for ΔNΔRep. In each case, two independent clones were characterized. Correct integration and replacement of *csp* with *Δrep* was confirmed by Southern blotting, PFGE and PCR (Figure S1). Replacement of wild type *csp* with *ΔnΔrep* was confirmed by PCR and sequencing of the resulting *csp* gene (Figure S2). The CSP sequence in both mutants is compared to wild type in Figure S3.

Both mutants were analyzed by Western blot for CSP expression using antisera specific for either: the NH_2_-terminus [9], the repeat region [25] or the COOH-terminus [9], [15] (Figure 1B). Three different control parasites were used: wild type *P. berghei* (WT), wild type *P. berghei* expressing GFP (WT-GFP) and a previously published *P. berghei* CSP recombinant control, RCon in which a wild type copy of *csp* was transfected into the *csp* locus [15]. Although the predicted molecular weight of CSP is 35 kDa, the full-length protein migrates anomalously due to the repeat region [15]. Thus, without the repeats, CSP fragments migrate closer to their expected molecular weight. Because the ΔRep and ΔNΔRep CSP would be expected to migrate at 20 kDa and 12 kDa respectively, we used 18% gels in order to resolve these mutant CSPs with the result that the higher molecular weight range was not as well resolved. As shown in Figure 1B (left panel), polyclonal antisera specific for the CSP NH_2_-terminus recognizes the full-length form of CSP but not the processed form, in all 3 control lines. In ΔRep parasites, it recognizes a single band, which is significantly smaller due to the absence of the repeats and in ΔNΔRep parasites no band is recognized. When the repeat region is deleted, as with the ΔRep parasites, the resulting protein migrates at the expected size of 20 kDa. Probing with a monoclonal antibody specific for the *P. berghei* repeats, mAb 3D11, confirms that the repeats are only present in wild type parasites and are not present in ΔRep and ΔNΔRep parasites (Figure 1B, middle panel). This was also confirmed by IFA (data not shown). Finally, probing with polyclonal antisera specific for the COOH-terminus of CSP showed the expected full-length and processed forms in control and ΔRep parasites but only one band in ΔNΔRep parasites (Figure 1B, right panel).

### ΔRep and ΔNΔRep parasites produce normal numbers of oocysts but have defects in sporozoite development

ΔRep and ΔNΔRep parasites developed normally during blood stages, forming gametocytes that appeared normal (data not shown). This was expected as there is little to no CSP expressed in blood stages and previous CSP deletion mutants had no blood stage phenotype [13], [14].

**Figure 2:**
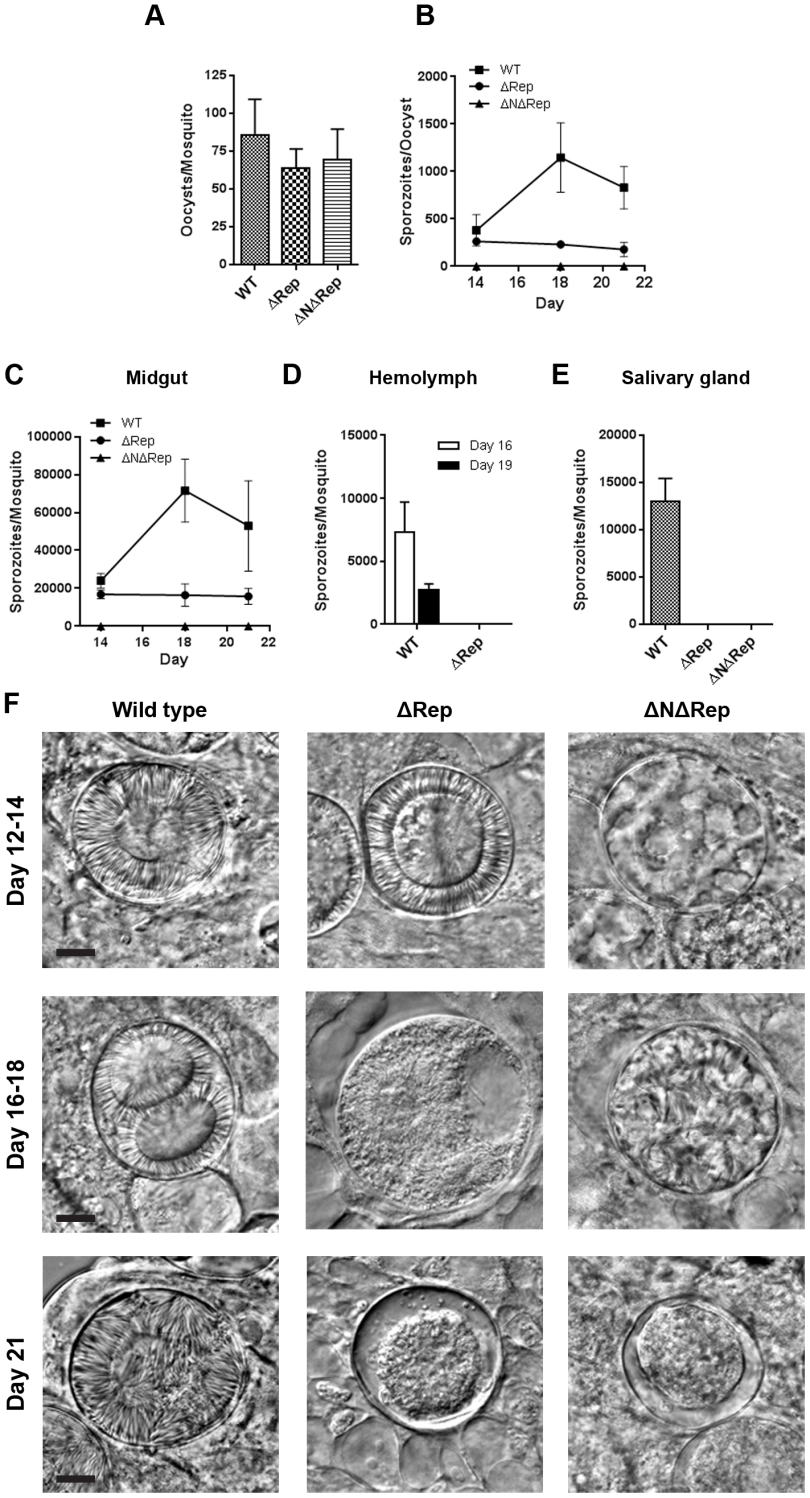
Phenotypic analyses of ΔRep and ΔNΔRep mutants in the mosquito. **A.** Oocyst numbers. On day 14 post-infection, midguts from 20–30 mosquitoes were scored for number of oocysts by phase or fluorescence microscopy. Shown is the mean ± SEM for each line. This analysis was performed 3 times with different batches of mosquitoes and a representative experiment is shown. **B.** Number of sporozoites per oocyst. On the indicated day post-infective blood meal, equal numbers of 10–20 mosquito midguts were collected and used either to count oocysts or were homogenized and sporozoites were counted. The number of sporozoites was then divided by the number of oocysts. Each point represents the mean ± SEM of 4 independent experiments. ΔNΔRep parasites did not produce sporozoites. **C.** Midgut sporozoite numbers. At each of the indicated days post-infective blood meal, midguts were dissected from 10–20 mosquitoes per parasite line, sporozoites were counted and the number of sporozoites per mosquito was calculated. Shown is the mean ± SEM of pooled data from 4 independent experiments. No sporozoites could be detected by light microscopy in the ΔNΔRep line. **D.** Hemolymph sporozoite numbers. On days 16 and 19 post-infective blood meal, hemolymph was collected from 15 mosquitoes and sporozoites were counted. Shown is the mean ± SEM of three independent experiments. No hemolymph sporozoites were observed in ΔRep infected mosquitoes. **E.** Salivary gland sporozoite numbers. On day 21 post-infective blood meal, salivary glands from 20 mosquitoes were dissected and sporozoites were counted. Shown is the mean ± SEM of 3 independent experiments. No salivary gland sporozoites were ever observed in ΔRep and ΔNΔRep infected mosquitoes. **F.** Representative differential interference contrast (DIC) microscopy images of oocysts from wild type, ΔRep and ΔNΔRep infected mosquitoes at the indicated days post infection. Bars represent 10 µm.

To follow parasite development in the mosquito, female *Anopheles* mosquitos were fed on mice infected with either the respective wild type or mutant parasites. On day 14 after an infected blood meal, oocyst numbers were determined. In more than 3 independent experiments from our two independent laboratories, there was no significant difference in oocyst number between wild type and mutant parasites (Figure 2A). Following this we looked at sporozoite development, beginning at days 13–14 post-infective blood meal when sporozoites are first visible. We quantified midgut sporozoites and also looked at oocysts by phase or fluorescence microscopy depending on the parasite line. At this time, no significant differences were observed between ΔRep and control parasites (Figure 2B, C and F). However, in mosquitoes infected with ΔNΔRep parasites, no sporozoite development in oocysts could be observed by light microscopy (Figure 2F) and we saw no sporozoites in homogenized midguts (Figure 2B and C; Table S1).

In control parasites we typically observe an increase in the number of midgut sporozoites between days 14 and 18 post-infective blood meal as sporogony proceeds. At approximately day 16, mature sporozoites begin to exit the oocyst and enter the hemocoel, leading to a decrease in midgut sporozoite numbers. Although ΔRep parasites looked normal at day 14, the sporozoite numbers did not increase as time went on (Figure 2C). To determine whether this was a defect in sporozoite development or whether sporogony occurred in fewer oocysts, we compared the number of sporozoites per oocyst in ΔRep and control parasites. As shown in Figure 2B, the initial number of sporozoites per oocyst in control and ΔRep parasites is comparable, however at later time points the number of sporozoites per oocyst increases in the control parasites, but not in ΔRep parasites, suggesting that although sporogony begins in a canonical fashion in the ΔRep mutant, it does not proceed normally. Observation of oocysts of the ΔRep mutant by phase-microscopy suggests that they were degenerating (Figure 2F, days 16–18 and day 21). Nonetheless, at day 21, morphologically normal sporozoites could be isolated from ΔRep-infected midguts by homogenization all be it at significantly lower numbers compared to controls (Figure 2B and 2C). Oocyst viability is described in more detail below. Although low numbers of ΔRep oocyst sporozoites were observed, we never detected ΔRep sporozoites in the hemolymph or in salivary glands (Figure 2D and E).

### Ultrastructural analysis of ΔRep and ΔNΔRep mutants show defects in sporozoite development

**Figure 3:**
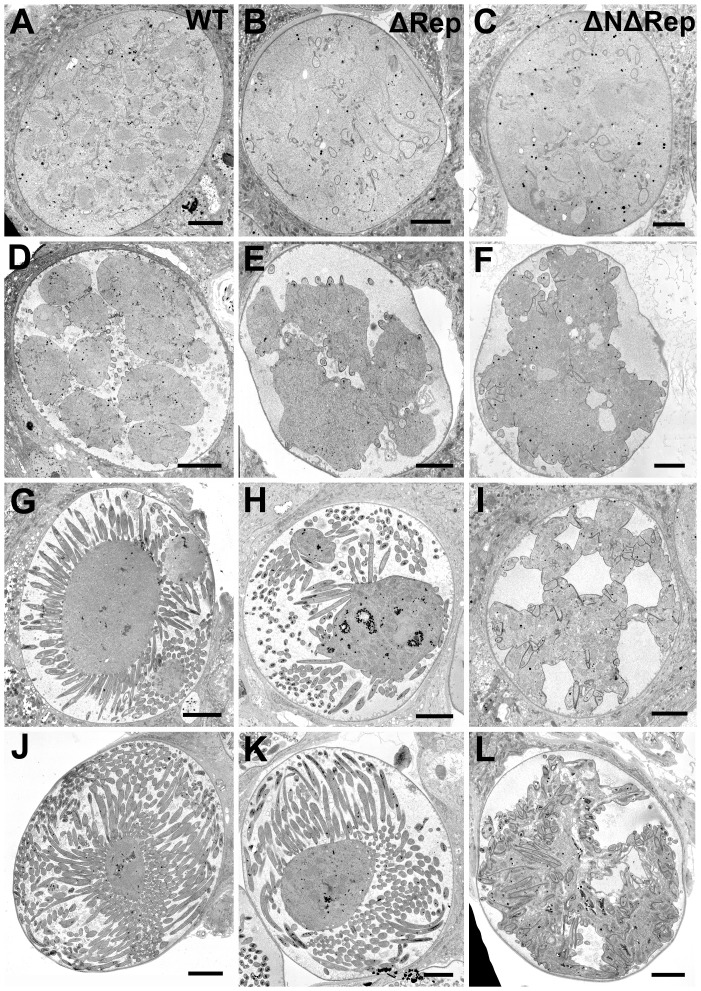
Electron micrographs of sporogony in WT, ΔRep and ΔNΔRep mutants. A series of electron micrographs of oocysts illustrating the progressive stages in the sporogonic process undergone by WT, ΔRep, and ΔNΔRep oocysts in the mosquito midgut. The structure of the oocysts at the end of the growth phase was similar for WT (**A**) and both mutants. (**B, C**) The initiation of sporozoite formation with retraction of the plasmalemma was also similar (**D-F**). However, while sporozoite formation continued by a budding process in both WT (**G**) and the ΔRep mutant (**H**) there was no budding seen in the ΔNΔRep mutant (**I**). This budding process continued until the sporozoites were fully formed in the WT (**J**) and ΔRep (**K**). In contrast the mature oocyst of the ΔNΔRep mutant contained a tightly adhered mass of sporozoites (**L**). Bars represent 10 µm.

Since the experiments detailed above demonstrated that the repeat mutants were arrested during the oocyst stage, we performed electron microscopy of wild type (WT), ΔRep and ΔNΔRep oocysts at different time points after the infective blood meal. An overview of oocyst growth and sporozoite development in each of the parasite lines is shown in Figure 3. As previously described [14], [26], [27] and shown in our analysis of wild type parasites (Figure 3A, D, G and J), initial growth of the oocyst is followed by a differentiation phase in which the progressive formation of the sporozoites from the cytoplasmic mass of the oocyst occurs. This process does not proceed normally in either of the two mutants, however, ΔNΔRep parasites are significantly more affected than ΔRep parasites (Figure 3).

The initial growth phase of oocysts was similar for WT and mutant parasite lines (Figure 3A-C). Following this, the differentiation phase begins with the focal retraction of the plasmalemma from the oocyst wall. This proceeds to deep invagination of the plasmalemma, which in many cases results in division of the original single cytoplasmic mass into multiple sporoblasts. Retraction of the plasma membrane occurs in both mutants however division of the cytoplasmic mass into individual sporoblasts does not appear to be complete and is most affected in the ΔNΔRep mutant (Figure 3D-F).

**Figure 4:**
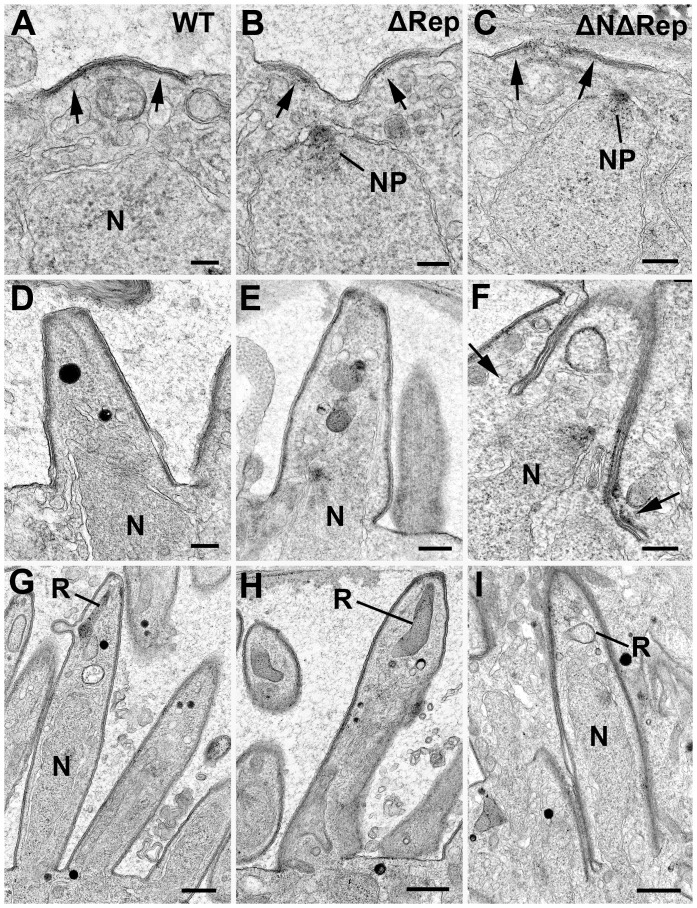
Electron micrographs of sporozoite formation in ΔRep and ΔNΔRep mutants. Details from the surface of the cytoplasmic mass of the oocysts: comparing WT sporozoite formation to ΔRep and ΔNΔRep mutants. **A, B, C.** Images showing the initiation of sporozoite development with the formation of flattened vacuoles forming the IMC beneath the plasmalemma (arrows) which is similar in WT (**A**) and both mutants (**B, C**). N – nucleus; NP – nuclear pole. Bar represents 100 nm. **D, E, F.** Images illustrating the early stages of sporozoite formation. Note the budding of the apex of the sporozoite from the cytoplasmic mass in both the WT (**D**) and ΔRep (**E**). In contrast, the IMC and plasmalemma show invagination in the ΔNΔRep (arrows) (**F**). N – nucleus. Bars represent 100 nm. **G, H, I.** Mid stage of sporozoite formation showing the continued budding from the surface and the appearance of rhoptry anlagen (R) in both WT (**G**) and ΔRep (**H**). In contrast, the growth of the ΔNΔRep sporozoites appeared to involve further invagination into the cytoplasmic mass (**I**). N – nucleus. Bars represent 500 nm.

Simultaneous with plasmalemma retraction, the first morphological evidence of initiation of sporozoite formation occurs, with focal deposition of flattened vacuoles beneath the plasmalemma leading to the development of the inner membrane complex (IMC) and sporozoite budding. Low power images show that these form at multiple distinct sites around the plasmalemma in both wild type and mutant parasites (Figure 3D-F). More detailed images in Figure 4A-C show the distinct localization of the IMC. IMC initiation is accompanied by the close apposition of a nucleus with a previous formed nuclear pole directed towards the IMC/plasmalemma (Figure 4B and C).

The ultrastructural features of sporozoite initiation were similar in the WT and both mutants (Figure 4A-C).

In both the WT and ΔRep mutant, sporozoite formation progresses with growth of the IMC, which immediately forms a cone-like protuberance representing the apex of the sporozoite: consisting of the plasmalemma, IMC and sub-pellicular microtubules forming a rigid scaffold (Figure 4D and E). The developing sporozoites appear to bud out from the cytoplasmic mass by growth of the cone-like structure forming the characteristic pellicle of the motile stages in both WT and ΔRep mutant (Figure 4D and E). As this growth occurs, the rhoptry anlagen appears in the apical region and, as budding proceeds, a nucleus followed by a mitochondrion and apicoplast enter the developing sporozoite (Figure 4G and H). This elongation continues until the sporozoite is fully formed including the maturation of the apical organelles (rhoptries and micronemes) (Figure 4G, H and I). At 18 and 21 days post infection it was possible, by electron microscopy to identify free sporozoites in the surrounding mosquito tissue in the wild type infections but no free sporozoites were observed in ΔRep mutant by electron microscopy.

**Figure 5:**
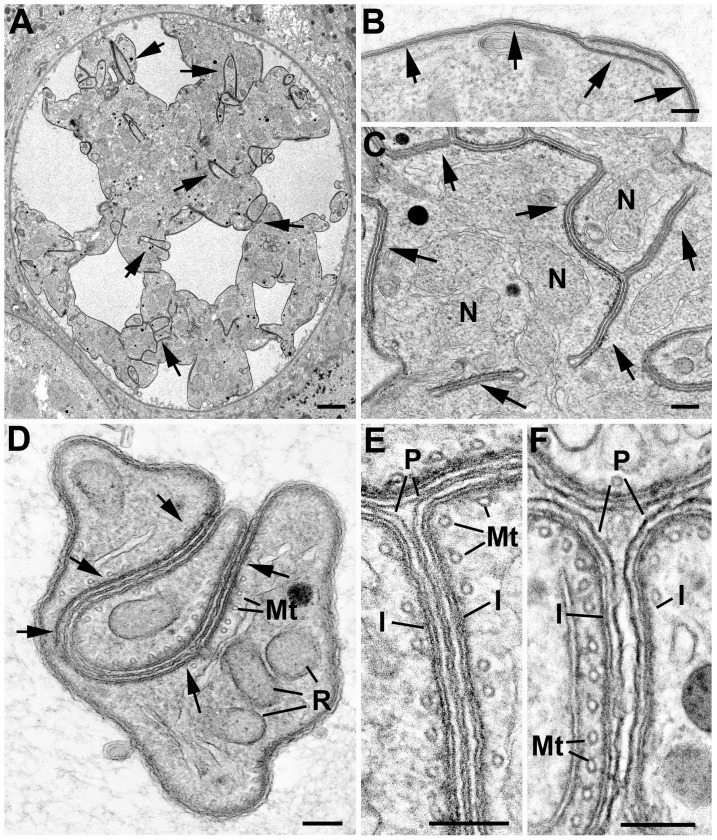
Electron micrographs showing unusual aspect of inner membrane complex development of the ΔNΔRep mutant. **A.** Low power of a mid-stage oocyst showing the retracted plasmalemma and areas of IMC invagination into the cytoplasmic mass (arrows). Bar is 1 µm. **B.** Detail of the surface of an early oocyst showing extensive growth of the IMC (arrows) but no evidence of budding. Bar is 100 nm. **C.** Detail of a more advanced stage in development showing areas of abnormal IMC/plasmalemma formation and invagination into the cytoplasmic mass of the sporoblasts (arrows). Bar is 100 nm. **D.** Cross section through two sporozoites showing loss of shape, adhesion, and folding of the plasmalemma of the sporozoites (arrows). R – rhoptry; Mt - microtubule. Bar is 100 nm. **E, F.** Enlargement of cross sections through ΔNΔRep (E) and WT (F) parasites, showing the relative distance between the plasmalemma of adjacent sporozoites. Note in the ΔNΔRep mutant the plasma membranes appeared tightly adhered (similar to that between the IMC membranes) (**E**) compared to the significantly wider space observed in the WT (**F**). I – IMC; Mt - sub-pellicular microtubules; P – plasmalemma. Bar is 100 nm.

In the ΔNΔRep mutant, while the initiation of sporozoite formation was similar, subsequent development was markedly abnormal (Figure 3I and L, Figure 4A-C, Figure 5). Rather than budding out from the cytoplasmic mass, sporozoite formation appeared to present multiple abnormalities. Unlike the WT and ΔRep where the IMC/plasmalemma immediately starts to bud outwards, the ΔNΔRep mutant exhibits extended lateral growth of the IMC along the plasmalemma (Figure 5B). There were also areas showing disorganized invaginations of the plasmalemma/IMC (Figure 5C). However, in few cases, the normal cone-like structure of the sporozoite consisting of the plasmalemma, IMC and sub-pellicular microtubules was observed (Figure 4F). Moreover rather than budding outwards, as sporozoite formation continued, the few normal shaped sporozoites appeared to grow by an invagination of the sporozoite membrane complex (plasmalemma, IMC and microtubules) into the cytoplasmic mass (Figure 4F and I, Figure 5A). As this occurred, there appeared to be tight adhesion between the adjacent areas of the plasmalemma. When examined in detail, the space between the adjacent regions of the plasmalemma was similar to that between the membranes of the IMC complex, measuring ∼5 nm (Figure 5E). Such close apposition was never seen in WT or ΔRep sporozoites, even in the most densely packed regions the space was in excess of 20 nm (Figure 5F). This resulted in tight adhesion between the plasmalemmas of adjacent sporozoites or with the plasmalemma of the residual oocyst in areas with underlying IMC (Figure 4F and I, Figure 5B and C). Tight adhesion between plasmalemmas was only seen where there was IMC underlying both plasmalemmas. Ultimately, only low numbers of sporozoites retained their shape and developed the same apical and cytoplasmic organelles as WT. In addition to abnormally-budding sporozoites, there were areas that appeared to lack a rigid scaffold with either loss of the sub-pellicular microtubules or disorganization of the microtubules, allowing pleomorphic folding of the pellicle around adjacent sporozoites with tight adhesion between the plasma membranes (Figure 5D). No fully formed or free sporozoites were observed and over time there was increasing evidence of degenerative changes both in the developing sporozoites and cytoplasmic mass.

This abnormal development of ΔNΔRep sporozoites appears to result from two separate factors. The first is a loss in the majority of sporozoite anlagen of the coordinated development of the components of the pellicle to form the rigid scaffold that maintains the shape of the sporozoites. This appears to be associated with misplacement or absence of the sub-pellicular microtubules resulting in disorganised IMC formation. The second is the apparent adhesion of adjacent areas of the plasmalemma resulting in an agglutinated mass of abnormal structures. Even the few sporozoites that do form are trapped by adhesion to adjacent structures.

### Abnormal sporozoite development ultimately leads to oocyst degeneration and parasite death

**Figure 6:**
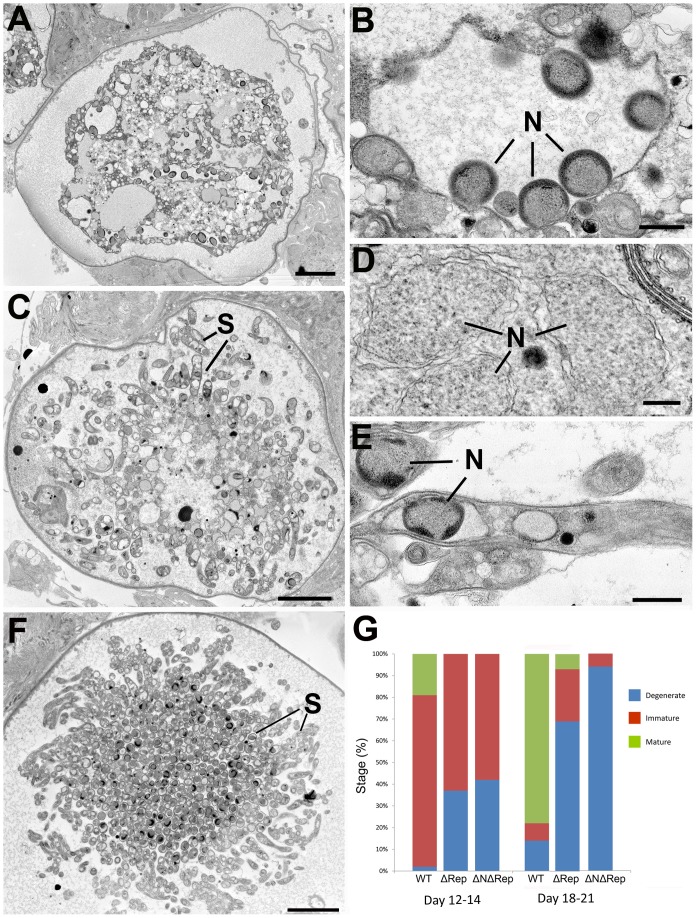
Electron micrographs illustrating the process of cell death observed in oocysts of the ΔRep and ΔNΔRep mutants. **A.** Low power of a ΔNΔRep oocyst with a degenerating, undifferentiated central cytoplasmic mass. Bar is 10 µm. **B.** Detail from the degeneration of a ΔNΔRep oocyst similar to that in **A** showing a dilated nuclear membrane containing a number of nuclei (N) exhibiting peripheral chromatin condensation. Bar is 100 nm. **C.** Low power of a ΔRep oocyst in which sporozoite formation had occurred but now exhibited features of cell degeneration. Bar is 10 µm. **D.** Detail of the nuclei observed in an intact ΔNΔRep oocyst showing the absence of any peripheral nuclear condensation. Bar is 100 nm. **E.** Longitudinal section through a sporozoite showing the nucleus with peripheral chromatin condensation and dilatation of the nuclear membranes. N – nucleus. Bar is 500 nm. **F.** Low power of a ΔRep oocyst in which there is a cross section of a central mass of degenerating sporozoites (S). Bar is 10 µm. **G.** Histogram comparing the relative number of immature mature and degenerate oocysts at two time points (12–14 days and 18–21 days) for WT, ΔRep and ΔNΔRep oocysts. (Based on EM examination of multiple midguts from multiple experiments – number of oocysts evaluated: 405 wild type; 236 ΔRep mutant; 165 ΔNΔRep mutant).

To quantitatively examine oocyst development, the relative numbers of immature, mature and degenerating oocysts were quantified by electron microscopy in wild type and ΔRep and ΔNΔRep mutants during two time periods corresponding to early and late stages of sporozoite development (Figure 6G). Immature oocysts were classified as those in which the sporozoite budding process had not been completed while mature oocysts were those in which fully formed sporozoites could be identified. Oocysts were classified as degenerating if they had the following features: vacuolated cytoplasm, peripheral chromatin condensation, abnormal multi-membranous structures, and/or swelling of sporozoites as described below. At the early time points, all three parasite lines showed a majority of immature oocysts, however, even at this time, the ΔRep and ΔNΔRep had a larger proportion of degenerating oocysts compared to wild type (Figure 6G). At the later time points, between day 18 and 21 post-infective blood meal, the majority of wild type oocysts were mature, whereas only a few intact mature oocysts were observed in ΔRep parasites and none were seen in ΔNΔRep parasites (Figure 6G). In both ΔRep and ΔNΔRep the vast majority of oocysts exhibited degenerative changes suggesting parasite death. This also reflected what was observed by light microscopy for WT and ΔRep (Figure 2).

When the dead and dying oocysts were examined by electron microscopy, distinctive features could be identified. While a number of dead oocysts were in an advanced stage of degeneration with an extensively vacuolated cytoplasm, others exhibited identifiable features. In the wild type and both mutants it was possible to identify characteristic degenerative changes that were present in both early oocysts (single cytoplasmic mass; Figure 6A) and oocysts where varying degrees of sporozoite formation had occurred (Figure 6C and F). The most marked morphological change was in the nucleus, which showed peripheral chromatin condensation that is a feature consistent with an apoptotic mechanism of cell death [28]. In both the polyploid nuclei within the cytoplasmic mass and nuclei within the partially formed sporozoites, there appeared to be significant peripheral chromatin condensation compared to normal nuclei with dispersed chromatin (compare Figure 6B and E with D). An unusual observation was that chromatin condensation occurred within the enlarged nucleus of the cytoplasmic mass, resulting in multiple individual nuclei forming within the outer nuclear membrane but with each individual nucleus being enclosed by the inner nuclear membrane (Figure 6B). Similar chromatin condensation was observed in oocysts containing sporozoites (Figure 6C and F). The degeneration continues with dilatation of the nuclear membrane (Figure 6B and E) and the rough endoplasmic reticulum plus cytoplasmic vacuolation forming multi-membranous structures. This combines with swelling of the mitochondria and apicoplasts and probably leads to the eventual lysis of the plasmalemma of both sporoblasts and sporozoites. There was also general swelling and loss of shape of the sporozoites. The process appeared similar in wild type and both mutants although the degenerative process appeared to be initiated earlier in the ΔNΔRep mutant than the wild type and ΔRep mutant where a proportion of the oocysts had advance sporozoite formation prior to the initiation of cell death. Oocyst death did not appear to result in any host tissue reaction and no melanisation was observed.

**Figure 7:**
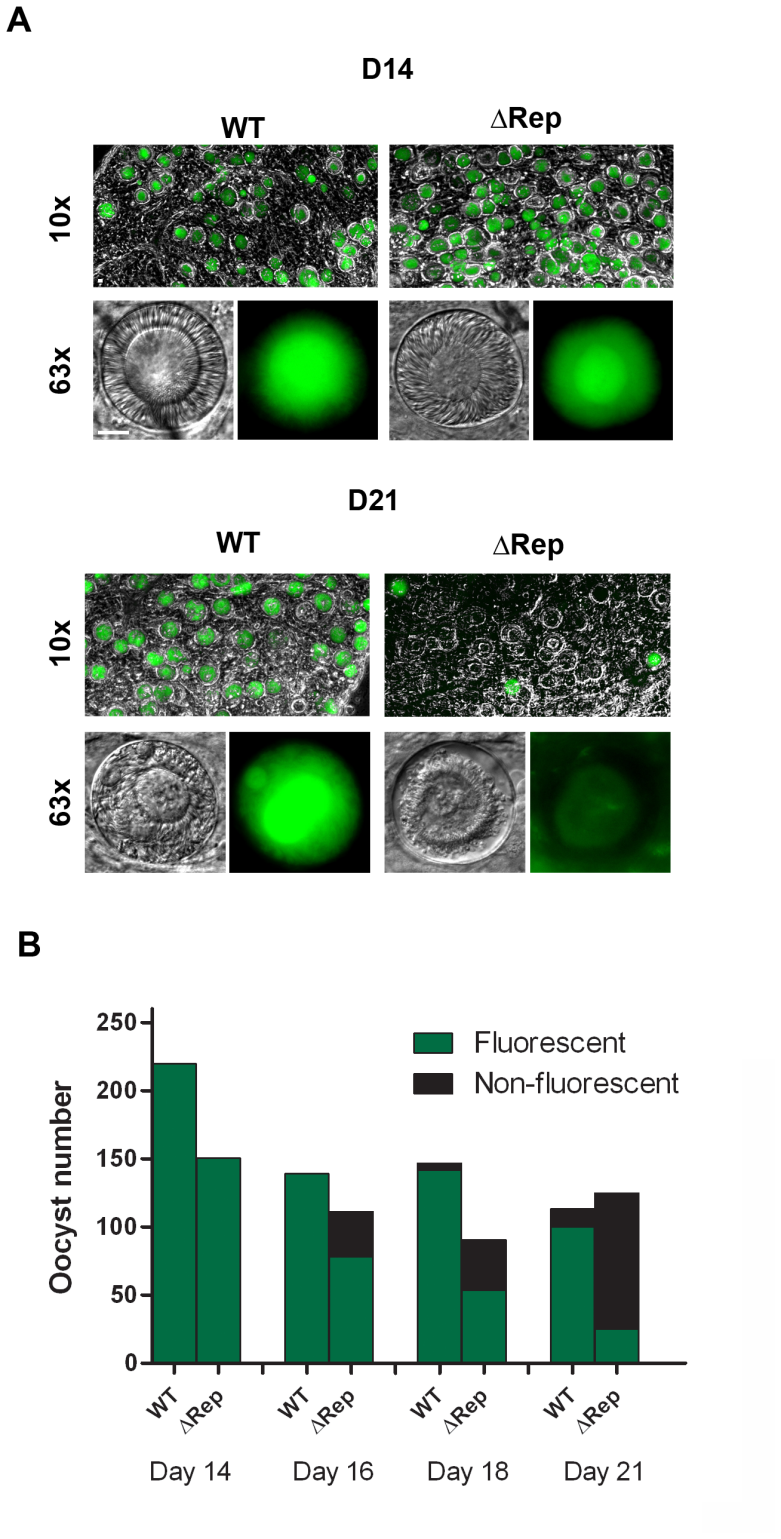
Light microscopy analysis of cell death in ΔRep oocysts. **A.** Photographs of representative mosquito midguts on days 14 and 21 post infection. Control and ΔRep parasites express GFP in most oocysts at day 14 post infection whereas by day 21 most of the ΔRep oocysts have lost the GFP fluorescence. Top panel shows a mosquito midgut and lower panel shows representative oocysts at higher magnification with degenerated features and absence of GFP fluorescence due to loss of plasmalemma integrity at day 21 post infection in ΔRep oocysts. **B.** Quantification of GFP positive and GFP negative oocysts at 14, 16, 18, and 21 days post infection for control and ΔRep oocysts.

Oocyst viability was further confirmed by scoring the number of GFP-positive oocysts in the ΔRep and control parasites. Most of the oocysts were GFP positive in ΔRep mutant at day 14 post-infection, but over time there was a loss of GFP positive oocysts. By day 21 post infection, 60–70% of ΔRep oocysts had lost GFP fluorescence and hence were no longer viable with concomitant loss of plasmalemma integrity (Figures 6 and 7). This loss of GFP coincided with the observation of degenerating oocysts from day 16–21 post infection (Figure 6G, Figure 7).

In the case of ΔNΔRep parasites, we never observed midgut, hemolymph or salivary gland sporozoites (Figure 2B, C and E) and similar to ΔRep parasites we observed a large number of degenerating oocysts at later time points (Figure 6G). Consistent with these findings neither ΔRep nor ΔNΔRep-infected mosquitoes could initiate a blood stage infection in naïve mice (Table S2).

## Discussion

Here we provide the evidence for the critical role of the repeat region of CSP in sporozoite development. We generated two transgenic *P. berghei* parasite lines expressing mutant forms of CSP; one lacked the central repeat region (ΔRep) and the other lacked both the NH_2_-terminal domain and the repeat region (ΔNΔRep). In both mutants we observed that the deletion of the repeats did not affect early stages of oocyst formation and growth. Following this, however, sporozoite development was significantly affected in the ΔNΔRep mutant with no free sporozoites being produced. In contrast, the ΔRep mutant showed normal sporozoite development at early stages of sporogony but development did not continue and oocysts degenerated.

Previous studies have demonstrated that CSP plays a central role in sporozoite development [13], [14]. By immune-electron microscopy, CSP is visible in oocysts by day 5 or 6 post blood meal, where it localizes to the oocyst plasma membrane and cytoplasm. Following this the IMC is laid down at discrete locations under the oocyst plasma membrane as it begins to retract from the oocyst capsule. As more IMC is laid down with associated microtubules under the cytoplasmic side of the plasma membrane of the sporoblast, sporozoite budding begins. Further growth and budding of the sporozoite continues with the extension of the plasma membrane, IMC and microtubules thus forming the triple membrane pellicle structure of the nacent sporozoite [26]. In the CSP knockout mutant (CSKO), the IMC is not restricted to small areas of the oocyst plasma membrane but is deposited extensively along the plasmalemma and subsequent sporoblast formation does not go to completion [14]. Normal sporozoite budding does not occur in the CSKO and when it did occur, it was partial and not polarized but parallel to or inside the syncytial mass [14]. Similar features were also seen in another CSP mutant, CS-ΔGP1, in which a predicted GPI anchor signal peptide was deleted, further supporting CSPs role in IMC deposition and sporozoite formation [17]. In both the CS-ΔGP1 and CSKO mutants, the sporozoite budding sites and cytokinesis were severely affected [14], [17]. The phenotype of the ΔNΔRep mutant, the more severely affected of our two repeat-less mutants, is similar to that of the CSKO and CS-ΔGP1 mutants. Thus, deletion of the N-terminal domain and repeat region together gives rise to a CSP null phenotype but with the additional feature of tight adhesion between plasma membranes. Interestingly, deletion of the N-terminal domain or repeat region separately (ΔNFull in [15] and ΔRep in this study), do not lead to a CSP null phenotype. Thus, the presence of either the N-terminal domain or the repeat region, together with the C-terminal TSR is sufficient for sporozoite development.

A possible explanation for this is that though the TSR functions in sporozoite development, its adhesive capacity must be mitigated by the N-terminal domain and/or the repeat region. This is supported by our observation that in the ΔNΔRep parasite membranes are fusing at an early stage of budding, which did not occur in the CSKO or CS-ΔGP1 mutants. Additionally, a previously published mutant in which only the N-terminus was deleted (ΔNFull mutant), and the repeat region and TSR were intact exhibited enhanced sporozoite budding which appeared normal by electron microscopy, and produced 2 to 3 times more sporozoites compared to controls [15]. Since TSR domains in other proteins have been shown to function in cell adhesion and cell migration [29]–[31], these data raise the possibility that the TSR may function in sporozoite budding and that the repeat region performs a critical regulatory function in controlling the direction of budding, possibly by modifying the adhesive capacity of the TSR.

When only the repeat region is removed, as in the ΔRep mutant described herein, a similar developmental pattern to control parasites is initially observed: both in membrane morphology and IMC initiation in the early stages of sporogonic development with normal microneme and rhoptry formation. This suggests that the repeat region of CSP is not functionally involved in sporoblast formation or sporozoite development at early stages of sporogony. However sporozoite development and maturation at later stages was severely affected in ΔRep. These sporozoites undergo cell death and the nuclear material becomes condensed and exhibits apoptotic features. Similar nuclear changes are described in an earlier study of degenerating oocysts [32]. What leads to this is not clear and was different to the CSKO or other CSP mutants previously studied by transmission electron microscopy [14], [17].

One possibility is that that the repeats may fulfill a conserved structural role [33]. The absence of the repeat region in the ΔRep mutant may result in misfolding of CSP, which could affect sporozoite structural morphology at later stages of differentiation within the oocyst and hence both the oocyst, and its sporozoites, undergoing cell death. Another possibility, consistent with the hypothesis that repeat-less CSP is misfolded, is that the abundant amount of misfolded CSP on the sporozoite surface could affect the function of other sporozoite membrane proteins that are required for final maturation. Importantly the phenotype of the ΔRep mutant is distinct from previously published mutants that have a defect in sporozoite egress from the oocyst [18], [34]. Sporozoites in these mutants developed normally and sporozoite numbers in the oocyst continued to increase over time, as they were unable to exit.

Previous studies in which *P. berghei* parasites were engineered to express chimeric CSP with repeat regions from other *Plasmodium* species, found no effect on parasite development and viability [21]–[23] indicating that the repeat region, despite the variation in amino acid sequence, has a conserved structural/functional role. The studies we have performed here with two CSP mutants lacking the repeats support this hypothesis. Future studies elucidating the mechanisms by which CSP functions during sporozoite development, as well as the role of the central repeat region during infection of the mammalian host, are still necessary and of keen interest for future vaccine approaches.

## Material and Methods

### Ethics statement

All animal work at Nottingham has passed an ethical review process and was approved by the United Kingdom Home Office. Work was carried out in accordance with the United Kingdom ‘Animals (Scientific Procedures) Act 1986’ and in compliance with ‘European Directive 86/609/EEC’ for the protection of animals used for experimental purposes under a UK Home Office Project License (40/3344). Sodium pentabarbitol was used for terminal anesthesia and combination of ketamine followed by antisedan was used for general anesthesia. All animal work at John Hopkins was approved by the Johns Hopkins University Animal Care and Use Committee (protocol #M011H467), which is fully accredited by the Association for Assessment and Accreditation of Laboratory Animal Care. Avertin was used for general anesthesia and euthanasia was performed by cervical dislocation on anesthetized mice. At both institutions, all efforts were made to minimize animal suffering.

### Animals

Tuck's Original (Harlan) or Swiss Webster (Taconic) outbred mice were used for all transfections and mosquito infections and C57Bl6 (Harlan) mice were used for mosquito bite back experiments.

### Generation of Targeting Constructs

#### *ΔRep* Parasites

To generate a *Δrep* gene without the central repeat region, the sequences encoding the NH_2_-terminal and COOH-terminal fragments of CSP were amplified with overlapping primers and engineered restriction sites to join the two fragments. First a ∼1.6 kb NH_2_-terminal fragment of the *csp* containing the 5′UTR region (starting at 1276 bp downstream of the gene) and covering the NH_2_-terminal region of the protein (up to Region I, 92 aa) was amplified with primers PbCS-N1 (GGC*CCGCGG* GGTACCAAATATTATATG) and PbCS-N2 (GGCCCACCTGGCTGG*GG TTGTTTCAATTTATT) with *Sac*II and *Bst*XI restriction sites respectively; the *Bst*XI site was designed by introduction of a silent mutation so that it overlapped sequences encoding the NH_2_-terminus of the COOH-terminal protein fragment. The restriction sites in the primers are shown in italics and the overlapping region of the two fragments are underlined; the silent mutation introduced in the reverse primer (PbCS-N2) is marked with asterisk. The 3′ fragment (∼0.6 kb) of the gene containing the 3′UTR region (up to 299 bp downstream of the gene) with COOH-terminal region (containing TSR, 245–347 aa) was amplified with primers PbCS-C1 (CAACC*C* *CA GCCAGGTGG TAATAAC) and PbCS-C2 (GGC*AAGCTT* CGATATCGTCATAGCAAG) with *Bst*XI and *Hind*III sites respectively. The two PCR amplified fragments were purified, digested with respective restriction enzymes and joined together by three-way ligation into the pPCR-Script SK(+) vector digested with *Sac*II and *Hind*III restriction enzymes. The complete gene was sequenced for confirmation. The transfection plasmid was constructed for double homologous recombination to replace the native *csp* locus with a *Δrep* variant with its control elements and a selection cassette (Figure S1). The transfection plasmid (p*Δrep*) was built from pPfCSP construct previously described [12]. Briefly, the construct contained a 5′UTR of the *P. berghei csp* gene encompassing nucleotides 1–1130 immediately upstream of the *P. berghei csp* start codon. The mutated *P. berghei csp Δrep,* lacking the repeat region, and the 3′UTR region of *P. berghei csp* encompassing nucleotides 1–1150 downstream of its stop codon into which the DHFR-TS selectable marker cassette was inserted at its *Hind*III site (+302). The repeatless *P. berghei csp* gene (588 bp long) was amplified with primers PbRL-N (GGC*GAATTC*ATGAAGAAGT GTACCATTTTAG) and PbRL-C (GGC*GGATCC* TTAATTAAAGAATACTAATAC); the amplified fragment was digested with *Eco*RI and *Bam*HI and cloned in the respective sites in the pPfCSP vector replacing the *P. falciparum csp* gene to generate the *Δrep* construct (Figure S1A). Restriction enzymes *Apa*I and *Xba*I were used to release the linear transfection construct.

#### *ΔNΔRep* Parasites

The *ΔnΔrep csp* gene was generated using a PCR-based approach beginning with the previously published pCSRep ΔNfull plasmid in which the NH_2_-terminus after the signal sequence through to the end of Region I had been deleted [15]. Primers were designed to flank the central repeat region in order to delete this region and include restriction sites to yield fragments that when ligated made the desired *csp* deletion mutant. A 722 bp fragment, from the *Kpn*I site in the 5′UTR through the *csp* signal sequence, was amplified using forward primer P1 (5′-AAAAAAGGTACCAAATATTATATGC-3′; existing *Kpn*I site underlined) and reverse primer P2 (5′-AGAGCAGCTGTCCATATCCTGGAAGT AGAG-3′; introduced *Pvu*II site underlined). A 321 bp *csp* fragment, from the end of the repeat region through the stop codon, was amplified using forward primer P3 (5′-CCACAGCCACCCGGGAATAACAATAAC-3′; introduced *Sma*I site underlined) and reverse primer P4 (5′-GTTTATTTAATTAAAGAATACTAATAC-3′; existing *Pac*I site underlined). Both PCR products were gel purified using the QIAquick gel extraction kit (Qiagen) and then digested with the appropriate restriction enzymes and ligated overnight at 15°C using T4 DNA ligase. Because several ligation products were possible, we performed a PCR amplification to identify the correct ligation product with primers spanning the 1043 bp *Kpn*I-*Pac*I fragment. The *ΔnΔrep csp* gene was then cloned into pCR4-TOPO and sequenced. The *Kpn*I-*Pac*I fragment was then cloned into the transfection vector, pCSRep, replacing WT *csp*. After initial transfections failed because the repeat portion of CSP was re-inserted by the parasite during homologous recombination, the pCSRep vector was altered to include a longer, 1.5 kb 3′UTR. Since there was already 450 bp of 3′UTR in the transfection vector, we amplified from *P. berghei* ANKA gDNA, an additional 1235 bp fragment downstream of this using forward primer P5 (5′-ACAATATTATTTAAGGGAATTCTAA-3′; existing *Eco*RI site underlined) and reverse primer P6 (5′- CAGTGGAATTCTGAACTACCTG-3′; introduced *Eco*RI site underlined). The 1235 bp PCR product was gel purified and digested with the appropriate restriction enzymes before ligating into pCSRep which has a drug selection cassette consisting of the human DHFR gene flanked by the 5′ and 3′ UTRs of *P. berghei* DHFR-TS, followed by a *csp* cassette.

### Parasite Transfection

Recombinant mutant *P. berghei* ANKA strain parasites were generated by double homologous recombination in which the native *csp* locus was replaced by the mutant copy of *csp* with its control elements and a selection cassette. ΔRep mutants were made in *P. berghei* ANKA line 507cl1, which express a *gfp* transgene in the *p230p* locus [24] and ΔNΔRep mutants were generated in *P. berghei* ANKA wild type parasites. For ΔNΔRep transfection, *P. berghei* ANKA schizonts were collected from Wistar rats, Swiss Webster mice or TO mice, electroporated with 5–10 µg of DNA as previously outlined [24], injected into a Swiss Webster mouse or a TO phenylhydrazine treated mouse [35] and selected with pyrimethamine before cloning by limiting dilution in mice.

### Genotypic analyses of mutant parasites

ΔRep mutants were verified by pulse field gel electrophoresis (PFGE), Southern blot and PCR of the *csp* locus. For Southern blotting, genomic DNA was isolated from wild type and mutant parasites, digested with *Eco*RV and separated on a 0.8% agarose gel before transfer to a nylon Hybond N+ membrane (GE Healthcare). The blot was probed with a 1.1 kb fragment homologous to the 3′ UTR of the *csp* gene that was amplified by PCR (primers 3′UTR1CS 5′-ATAAACATTACGCATGATTAT-3′ and 3′UTR2CS 5′-GAGTACTCACGAATCCGAAATAAG-3′) and labeled using the AlkPhos direct labeling kit according to manufacturer's instructions (GE Healthcare). For pulsed field gel electrophoresis (PFGE) chromosomes of wild type and mutant parasites were separated on a CHEF DR III (BioRad) system using a linear ramp of 60–500 s for 72 h at 4 V/cm. The gel was blotted onto a nylon membrane and hybridized with a probe recognizing the resistance cassette in the targeting vector, the 3′ UTR of the *P. berghei* dhfr/ts locus on chromosome 7 and the GFP-expression cassette of reference line, 507cl1, that contains a single copy of *gfp* integrated into the *230p* locus [24]. Diagnostic PCR was performed to determine the deletion of the repeat region. The coding region of *csp* was amplified using the primers CSF (5′-ATGAAGAAGTGTACCATTTTAG-3′) and CSR (5′-TTAATTAAAGAATACTAATACTAA-3′). This resulted in a 1023 bp fragment for wild type *csp* and a 567 bp fragment for the *Δrep* mutant.

*ΔnΔrep* clones were verified by PCR for 3′ and 5′ integration as well as the *csp* coding sequence. PCR to verify integration at the *csp* locus was performed with primers DP1 (5′- AATGAGACTATCCCTAAGGG–3′) and DP2 (5′- TAATTATATGTTATTTTATT TCCAC-3′) for 5′ integration (1.1 kb product) and P7 (5–CGCCTGAGCAGCCTTTGTGT-3′) and P8 (5′–TCGAAATGGGCGCTGACAAGAA-3′) for 3′ integration (4.16 kb product). To amplify the *csp* locus primers P9 (5′- AGCACGCTTTTACTTTGTCCAGGT-3′) and P10 (5′- ACAAATCCTAATGAATTGCTTACA-3′) were used resulting in a 1976 bp fragment in WT *csp* and 1476 bp fragment for *ΔnΔrep csp*, in the latter case the sequenced was verified.

### Parasite development in the mosquito

*Anopheles stephensi* mosquitoes (3–6 day old) were allowed to feed on anesthetized mice infected with either wild type or mutant parasites at comparable numbers of gametocytes as assessed by blood smears. Mosquitoes were dissected on the indicated days post blood meal. For midgut and salivary gland sporozoites, organs from 20 mosquitoes were pooled and homogenized and released sporozoites were counted using a hemocytometer. Hemolymph from 15 mosquitoes was collected by perfusion of the thorax and abdomen with DMEM and sporozoites were counted as above. For oocyst counts, midguts were harvested, mounted on a slide and oocysts counted using phase or fluorescence microscopy. To quantify sporozoites per oocyst, the same number of mosquitoes from the same cage, was used for counting the number of oocysts and number of sporozoites. This varied among experiments but was between 10 and 20 mosquitoes for each quantification. Then the total number of sporozoites was divided by the total number of oocysts. For light microscopy photographs of developing oocysts, approximately 15–20 midguts were dissected from mosquitoes on the indicated days and mounted under Vaseline-rimmed cover slips. Images were taken with an AxioCam ICc1 digital camera fitted to a Zeiss AxioImager M2 microscope using a 63x oil immersion objective.

### Western blot

Midguts were dissected in RPMI with 1x protease inhibitors (Roche Complete e Mini Protease Inhibitors Cat. # 11836153001). Either sporozoites liberated from midguts or whole midguts (in cases where no sporozoites developed), were lysed in boiling sample buffer with 1x protease inhibitors and 50 mM DTT. For ΔRep and WT-GFP parasites, 10^4^ sporozoite equivalents were loaded per lane. For ΔNΔRep and RCon parasites, 1.5 whole midgut equivalents were loaded per lane for probing with NH_2_-terminal antisera [9] or 0.6 whole midgut equivalents were loaded per lane for probing with mAb 3D11, specific for the CSP repeats [25] and COOH-terminal antisera [9]. Samples were run on an 18% SDS-PAGE and transferred to a nitrocellulose membrane (BIO-RAD Cat. # 162-0112). The membranes were blocked with 2% milk, 3% BSA in Tris Buffered Saline pH 7.5, 0.1% Tween (TBS-T) and then incubated in primary antibodies diluted in 1% BSA/TBS-T (1 µg/ml 3D11, or a 1∶200 dilution of polyclonal NH_2_-terminal or COOH terminal antisera) followed by incubation in anti-mouse or anti-rabbit Ig conjugated to horseradish peroxidase. Bound antibodies were visualized using enhanced chemiluminescence (GE Healthcare).

### Electron Microscopy

The guts from mosquitos at 12–14 days post-infection and 18–21 day post-infection were dissected and fixed in 2.5% glutaraldehyde in 0.1 M phosphate buffer and processed for routine electron microscopy. This can be summarized as: samples were post-fixed in osmium tetroxide, dehydrate in ethanol, treated with propylene oxide and embedded in Spurr's epoxy resin. Thin sections were stained with uranyl acetate and lead citrate prior to examination in a Jeol 1200EX electron microscope.

## Supporting Information

Figure S1 **Generation and genotypic analysis of** ***Δrep*** **parasites.** **A.** Schematic representation of the endogenous *csp* locus, the targeting construct and the recombined *csp* locus following double cross-over recombination. Arrows P1 and P2 indicate PCR primers used to analyze the *csp* locus following recombination. *Eco*RV restriction sites and probe binding sites for Southern blotting are indicated. n: NH_2_-terminus, rep: repeat region, TSR thrombospondin repeat and COOH-terminus. **B.** Southern blot analysis of two independent *Δrep* clones and wild type parasite genomic DNA following *Eco*RV digest. A probe specific for the *csp* 3′UTR bound to 3.9 kb and 1.4 kb bands in wild type as expected and to 2.0 kb and 3.4 kb bands in *Δrep* parasites. **C.** Southern blot of Pulse Field Gel Electrophoresis (PFGE) using a *pbdhfr* 3′UTR probe. The probe recognizes the endogenous *dhfr* locus on chromosome 7, the *gfp* cassette integrated in the 230p locus of the GFP-transgenic parasites used for transfection (chromosome 4), and the recombined *csp* locus on chromosome 4 in *Δrep* parasites (cl. 5 and cl. 10). **D.** PCR of the *csp* locus in wild type and *Δrep* parasites. Wild type *csp* is 1 kb and *Δrep csp* is 0.6 kb.
(TIF)

Figure S2 **Generation and genotypic analysis of** ***ΔnΔrep*** **parasites.** **A.** Schematic representation of the endogenous *csp* locus, the targeting construct and the recombined *csp* locus following double cross-over recombination. The targeting construct contains 730 bp of *csp* 5′UTR (thick black line), the selectable marker *hdhfr* with its upstream and downstream control elements (grey box and thick grey lines) and the *csp* gene flanked by its upstream and downstream control elements (black box and thick black lines). It should be noted that 1.5 kb of 3′UTR was necessary to direct homologous recombination without concomitant correction of the introduced deletion. The plasmid containing the targeting construct, pCSRep, was digested with *Xho*I and *Kas*I to release the fragment for transfection. The dotted grey lines indicate the location of homologous recombination with the endogenous *csp* locus. Primers used for diagnostic PCRs are indicated on the recombined locus. n: NH_2_-terminus, rep: repeat region, TSR thrombospondin repeat and COOH-terminus. **B.** The results of diagnostic PCRs using genomic DNA from *ΔnΔrep* clones from two independent transfections (cl. 1 and cl. 2), recombinant control parasites (RCon) in which the transfection was performed with a full-length copy of *csp*, and wild type parasites (WT) are shown. In the left panel, 5′ integration is confirmed with primers DP1 and DP2 which amplify a 1.1 kb product from both *ΔnΔrep* clones and RCon parasites but not from WT. In the middle panel, 3′ integration is confirmed using primer P7 and P8 which amplify a slightly smaller product in the *ΔnΔrep* clones compared to RCon parasites because of the truncation of the *csp* gene: a 4.2 kb product is observed in the mutant parasites while a 4.8 kb product is seen in RCon parasites. In the right panel, amplification of the *csp* locus with P9 and P10, which also amplifies *csp* 5′UTR and a portion of the *hdhfr* 3′UTR, results in a 1.47 kb fragment in the mutant clones and a 1.97 kb fragment in WT parasites.
(TIF)

Figure S3 **Alignment of CSP from WT, ΔRep and ΔNΔRep Parasites.** Alignment was performed using ClusterW software. Region I is underlined for reference.
(PDF)

Table S1 **Raw data from 4 independent experiments used to generate** **Figure 2B and 2C** **.**
(PDF)

Table S2 **Infected mosquito bite experiment with WT, ΔRep and ΔNΔRep infected mosquitoes.**
(PDF)
