# Supplementary figures and images for: The Repeat Region of the Circumsporozoite Protein is Critical for Sporozoite Formation and Maturation in Plasmodium
Source: PLoS One. 2014 Dec 1;9(12):e113923. doi: 10.1371/journal.pone.0113923 (PMC4250072; doi:10.1371/journal.pone.0113923)

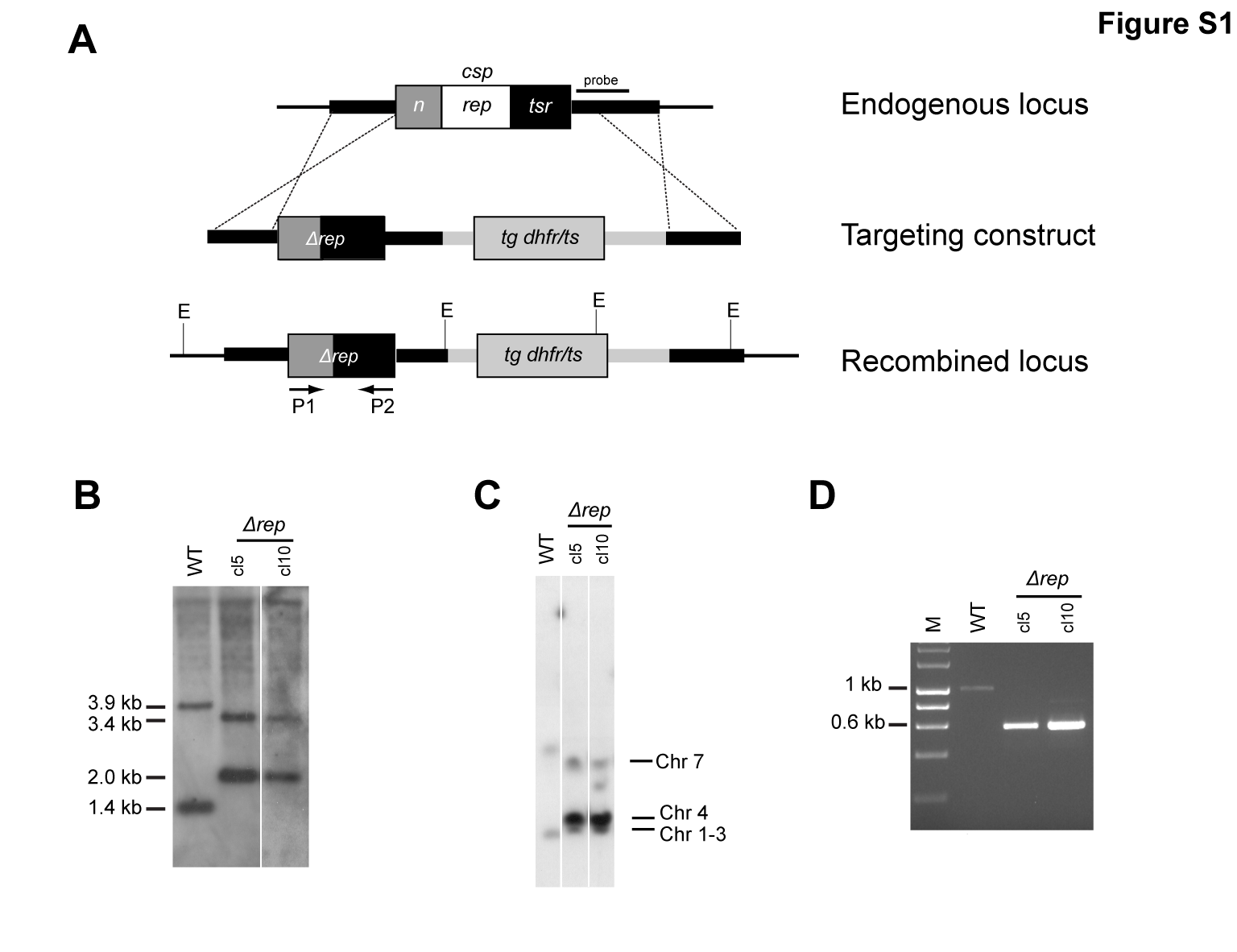

Supplement: Figure S1 — Generation and genotypic analysis of Δrep parasites. A. Schematic representation of the endogenous csp locus, the targeting construct and the recombined csp locus following double cross-over recombination. Arrows P1 and P2 indicate PCR primers used to analyze the csp locus following recombination. EcoRV restriction sites and probe binding sites for Southern blotting are indicated. n: NH2-terminus, rep: repeat region, TSR thrombospondin repeat and COOH-terminus. B. Southern blot analysis of two independent Δrep clones and wild type parasite genomic DNA following EcoRV digest. A probe specific for the csp 3′UTR bound to 3.9 kb and 1.4 kb bands in wild type as expected and to 2.0 kb and 3.4 kb bands in Δrep parasites. C. Southern blot of Pulse Field Gel Electrophoresis (PFGE) using a pbdhfr 3′UTR probe. The probe recognizes the endogenous dhfr locus on chromosome 7, the gfp cassette integrated in the 230p locus of the GFP-transgenic parasites used for transfection (chromosome 4), and the recombined csp locus on chromosome 4 in Δrep parasites (cl. 5 and cl. 10). D. PCR of the csp locus in wild type and Δrep parasites. Wild type csp is 1 kb and Δrep csp is 0.6 kb. (TIF) [file pone.0113923.s001.tif]

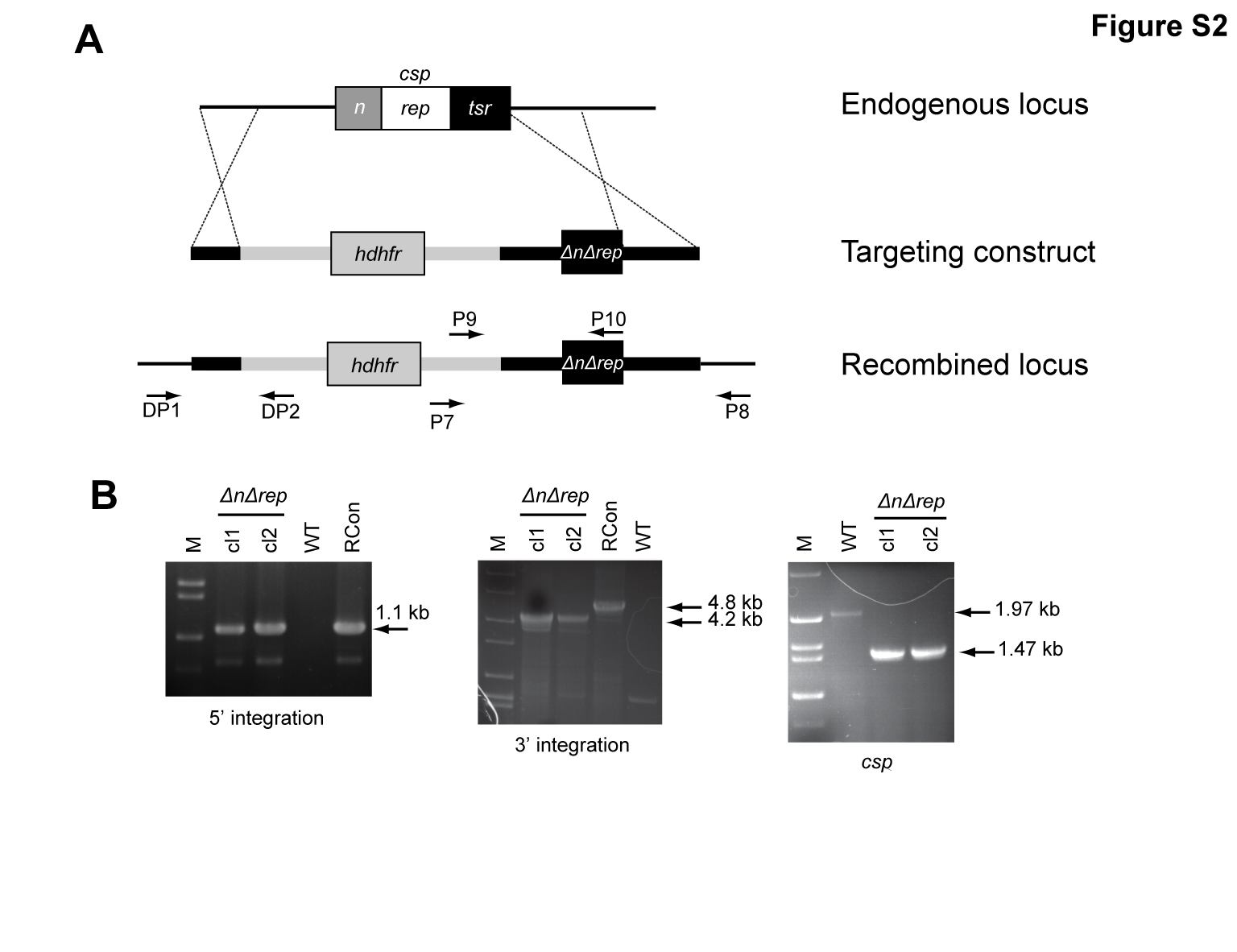

Supplement: Figure S2 — Generation and genotypic analysis of ΔnΔrep parasites. A. Schematic representation of the endogenous csp locus, the targeting construct and the recombined csp locus following double cross-over recombination. The targeting construct contains 730 bp of csp 5′UTR (thick black line), the selectable marker hdhfr with its upstream and downstream control elements (grey box and thick grey lines) and the csp gene flanked by its upstream and downstream control elements (black box and thick black lines). It should be noted that 1.5 kb of 3′UTR was necessary to direct homologous recombination without concomitant correction of the introduced deletion. The plasmid containing the targeting construct, pCSRep, was digested with XhoI and KasI to release the fragment for transfection. The dotted grey lines indicate the location of homologous recombination with the endogenous csp locus. Primers used for diagnostic PCRs are indicated on the recombined locus. n: NH2-terminus, rep: repeat region, TSR thrombospondin repeat and COOH-terminus. B. The results of diagnostic PCRs using genomic DNA from ΔnΔrep clones from two independent transfections (cl. 1 and cl. 2), recombinant control parasites (RCon) in which the transfection was performed with a full-length copy of csp, and wild type parasites (WT) are shown. In the left panel, 5′ integration is confirmed with primers DP1 and DP2 which amplify a 1.1 kb product from both ΔnΔrep clones and RCon parasites but not from WT. In the middle panel, 3′ integration is confirmed using primer P7 and P8 which amplify a slightly smaller product in the ΔnΔrep clones compared to RCon parasites because of the truncation of the csp gene: a 4.2 kb product is observed in the mutant parasites while a 4.8 kb product is seen in RCon parasites. In the right panel, amplification of the csp locus with P9 and P10, which also amplifies csp 5′UTR and a portion of the hdhfr 3′UTR, results in a 1.47 kb fragment in the mutant clones and a 1.97 kb fragment in WT parasites [file pone.0113923.s002.tif]
