# Supplementary material for: The Repeat Region of the Circumsporozoite Protein is Critical for Sporozoite Formation and Maturation in Plasmodium
Source: PLoS One. 2014 Dec 1;9(12):e113923. doi: 10.1371/journal.pone.0113923 (PMC4250072; doi:10.1371/journal.pone.0113923)
Supplement: Figure S3 — Alignment of CSP from WT, ΔRep and ΔNΔRep Parasites. Alignment was performed using ClusterW software. Region I is underlined for reference. (PDF) [file pone.0113923.s003.pdf]

Figure S3

```
WT          1 MKKCTILVVASLLLVNSLLPGYGQNKSIQAQRNLNELCYNEGNDNKLYHV  50
ΔRep        1 MKKCTILVVASLLLVNSLLPGYGQNKSIQAQRNLNELCYNEGNDNKLYHV  50
ΔNΔRep      1 MKKCTILVVASLLLVNSLLPGYGQ--------------------------  24

WT         51 LNSKNGKIYIRNTVNRLLADAPEGKKNEKKNKIERNNKLKQPPPPPNPND 100
ΔRep       51 LNSKNGKIYIRNTVNRLLADAPEGKKNEKKNKIERNNKLKQP--------  92
ΔNΔRep     25 --------------------------------------------------  24

WT        101 PPPPNPNDPPPPNPNDPPPPNPNDPPPPNPNDPPPPNANDPPPPNANDPA 150
ΔRep       93 --------------------------------------------------  92
ΔNΔRep     25 --------------------------------------------------  24

WT        151 PPNANDPAPPNANDPAPPNANDPPPPNANDPPPPNPNDPAPPNANDPPPP 200
ΔRep       93 --------------------------------------------------  92
ΔNΔRep     25 --------------------------------------------------  24

WT        201 NPNDPAPPQGNNNPQPQPRPQPQPQPQPQPQPQPQPQPRPQPQPQPGGNN 250
ΔRep       93 --------------------------------------------QPGGNN  98
ΔNΔRep     25 -----------------------------------------------GNN  27

WT        251 NNKNNNNDDSYIPSAEKILEFVKQIRDSITEEWSQCNVTCGSGIRVRKRK 300
ΔRep       99 NNKNNNNDDSYIPSAEKILEFVKQIRDSITEEWSQCNVTCGSGIRVRKRK 148
ΔNΔRep     28 NNKNNNNDDSYIPSAEKILEFVKQIRDSITEEWSQCNVTCGSGIRVRKRK  77


WT        301 GSNKKAEDLTLEDIDTEICKMDKCSSIFNIVSNSLGFVILLVLVFFN    347
ΔRep      149 GSNKKAEDLTLEDIDTEICKMDKCSSIFNIVSNSLGFVILLVLVFFN    196
ΔNΔRep     78 GSNKKAEDLTLEDIDTEICKMDKCSSIFNIVSNSLGFVILLVLVFFN    124
```
