# Supplementary material for: The Repeat Region of the Circumsporozoite Protein is Critical for Sporozoite Formation and Maturation in Plasmodium
Source: PLoS One. 2014 Dec 1;9(12):e113923. doi: 10.1371/journal.pone.0113923 (PMC4250072; doi:10.1371/journal.pone.0113923)
Supplement: Table S1 — Raw data from 4 independent experiments used to generate Figure 2B and 2C . (PDF) [file pone.0113923.s004.pdf]

Table S1

	WT			ΔRep			ΔNΔRep		
	**D13-14**	**D18-19**	**D21-22**	**D13-14**	**D18-19**	**D21-22**	**D14**	**D18**	**D21**
**Exp 1***	125	498	869	248	230	12.5	0	0	0
**Exp 2***	324	896	1400	195	187	314	0	0	0
**Exp 3***	861	2200	316	344	175	88	0	0	0
**Exp 4****	204	988	732	263	329	295			

Sporozoites per oocyst. At each of the indicated time points post infective bloodmeal, and for each of the indicated parasite lines, the number of sporozoites/oocyst is shown. This number was generated by dividing the average number of midgut sporozoites per mosquito by the average number of oocysts per mosquito. For Expts 1-3, 20 mosquitoes per point were used and for Expt 4, 10 mosquitoes per point were used.

	WT			ΔRep			ΔNΔRep		
	**D13-14**	**D18-19**	**D21-22**	**D14-13**	**D18-19**	**D21-22**	**D14**	**D18**	**D21**
**Exp 1**	23000	72320	85228	22100	13120	11500	0	0	0
**Exp 2**	13950	52000	11000	10952	7680	25200	0	0	0
**Exp 3**	26700	44000	13291	16000	10880	6300	0	0	0
**Exp 4**	32400	118000	102437	18450	34000	20000			

Sporozoites per midgut. At each of the indicated time points post-infective bloodmeal, and for each of the parasite lines, the average number of midgut sporozoites per mosquito was calculated. For Expts 1-3, 20 mosquitoes per point were used and for Expt 4, 10 mosquitoes per point were used.
