# Supplementary material for: The Repeat Region of the Circumsporozoite Protein is Critical for Sporozoite Formation and Maturation in Plasmodium
Source: PLoS One. 2014 Dec 1;9(12):e113923. doi: 10.1371/journal.pone.0113923 (PMC4250072; doi:10.1371/journal.pone.0113923)
Supplement: Table S2 — Infected mosquito bite experiment with WT, ΔRep and ΔNΔRep infected mosquitoes. (PDF) [file pone.0113923.s005.pdf]

Table S2

Strain	Mice positive / total mice	Prepatent period
WT	6/6	4
∆Rep	0/6	-
∆N∆Rep	0/6	-

Mouse infection after bite with WT, ΔRep and ΔNΔRep infected mosquitoes. Prepatent period is in days.
